# Progressively Enlarging Lip Swelling in an Indian Child Due to Entomophthorales Infection

**DOI:** 10.4269/ajtmh.24-0775

**Published:** 2025-05-20

**Authors:** Souradeep Chowdhury, Gagandeep Singh, Immaculata Xess

**Affiliations:** All India Institute of Medical Sciences, New Delhi, India

A 2-year-old male child from the state of Bihar, northern India, presented with swelling of the right cheek, which had initially started 1 year earlier and had gradually progressed to involve both lips and the perioral region. There was continuous dribbling of saliva, and he had been unable to take solid food orally for 2 months but was able to accept liquids.

**Figure 1.**
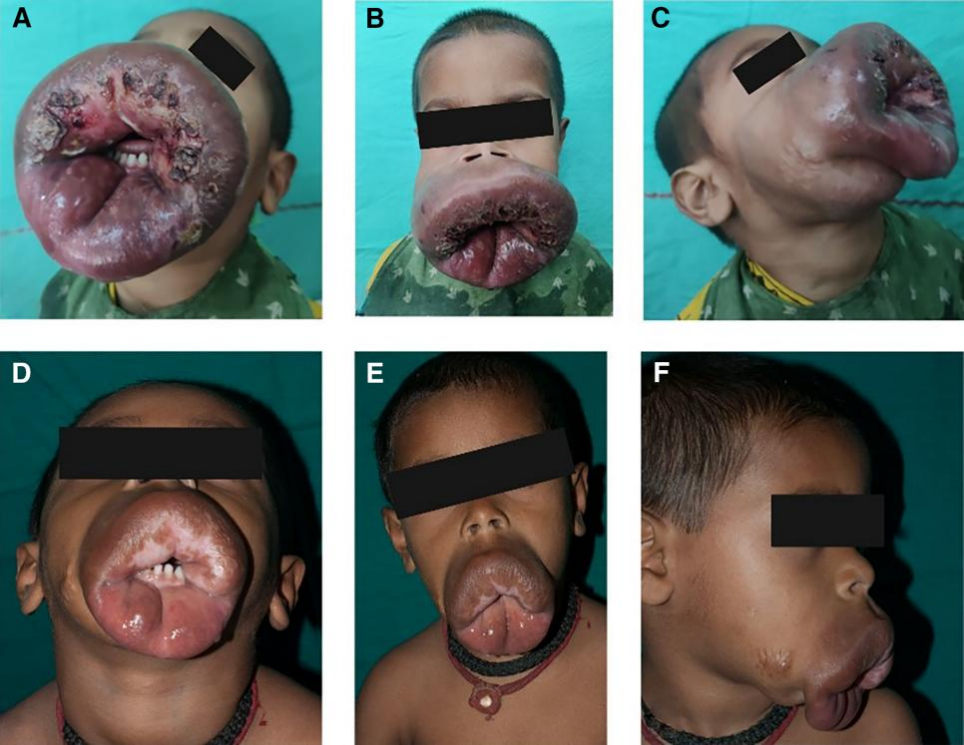
(**A**, **B**, and **C**) Circumferential involvement of the perioral and oral region, including the lips, with some extension into the bilateral malar and submental regions. A firm, nontender, noncompressible, multilocular subcutaneous swelling is present, exhibiting a dusky erythematous hue and measuring ∼10 × 9 cm^2^. (**D**, **E**, and **F**) Twelve months of treatment resulted in a significant reduction in the size, consistency, and swelling of the lesion.

On examination, his vitals were normal. There was circumferential involvement of the perioral and oral regions, including the lips, with some extension into the bilateral malar and submental regions. A firm, nontender, noncompressible, multilocular subcutaneous swelling with a dusky erythematous hue in places and measuring ∼10 × 9 cm^2^ was present (Figure 1). A clear margin existed between the normal and affected tissue, and the swelling could be lifted off deeper tissues via finger insinuation. The overlying skin was tense, edematous, and desquamating in places. A hemorrhagic scale crust covered underlying superficial erosions on the cutaneous aspect of the enlarged upper and lower lips. There were no palpable regional lymph nodes and no local rise in temperature or thrill over the swelling, which was causing significant morphological distortion.

**Figure 2.**
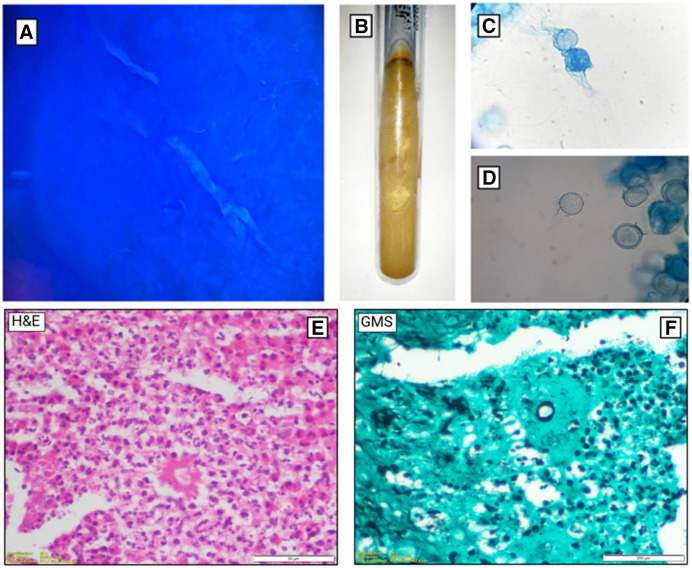
(**A**) Broad, pauci-septate hyphae seen with KOH and Calcofluor staining. (**B**) Culture of *Basidiobolus ranarum.* (**C** and **D**) Beaked zygospores seen in lactophenol cotton blue mount microscopy. (**E** and **F**) Splendore–Hoeppli phenomenon observed on an H&E stain and broad fungal hyphae observed on a special fungal stain—Grocott’s methenamine silver. H&E = hematoxylin and eosin; KOH = potassium hydroxide.

An initial suspicion of Entomophthorales infection was maintained. A skin biopsy from the lesion was sent for potassium hydroxide and Calcofluor staining, which revealed broad pauci-septate hyphae (Figure 2), suggestive of Entomophthorales. Treatment was initiated with a combination of saturated solution of potassium iodide and itraconazole syrup formulation. The sample was subsequently processed for both culture and histopathological examination. The culture was typical of *Basidiobolus ranarum* (Figure 2), and microscopy from a lactophenol cotton blue mount revealed characteristic beaked zygospores (Figure 2). Histopathology also revealed the Splendore–Hoeppli phenomenon on an hematoxylin and eosin stain, and broad fungal hyphae were observed on the special fungal stain, Grocott’s methenamine silver (Figure 2). Basidiobolomycosis is known to cause infections of the perineum, thighs, buttocks, thorax, or back. The striking aspect of this case is that although the affected body part is typically seen in Conidiobolomycosis, the identified etiological agent was *Basidiobolus*. The child was followed up at the Infectious Diseases clinic, where clinical response and serum levels of itraconazole were monitored. Recently, he completed 12 months of therapy, resulting in a significant reduction in both the size and consistency of the lesion (Figure 1). There is no residual nodularity of the swelling, and the patient is being considered for debulking and reconstruction.

A typical presentation of a rare disease was described in this article, and a high degree of clinical suspicion is required for the early diagnosis of such cases. The diagnosis highlights the need for a multidisciplinary approach to infectious diseases.

